# A Cross-Sectional Study Comparing AI-Generated Educational Content on Pneumonia for Medical Professionals With UpToDate

**DOI:** 10.7759/cureus.93984

**Published:** 2025-10-06

**Authors:** Aarushi Grover, Burhanuddin Kherodawala, Neha Uppal, Aaditya Jagadish, Khushboo Rani, Lekshmi Ravi

**Affiliations:** 1 Internal Medicine, Sri Ramachandra Institute of Higher Education and Research, Chennai, IND; 2 Pulmonology, Bombay Hospital, Mumbai, IND; 3 Gastroenterology and Hepatology, Shalamar Medical And Dental College, Lahore, PAK; 4 General Surgery, Northeast Georgia medical center, Gainesville, USA; 5 Internal Medicine, Goa Medical College and Hospital, Goa, IND; 6 Internal Medicine, North Alabama Medical Center, Florence, USA

**Keywords:** artificial intelligence, chatgpt, clinical decision support, educational content, medical education, pneumonia, uptodate

## Abstract

Introduction: Accurate and up-to-date educational resources are vital for medical professionals treating pneumonia to ensure alignment with evolving clinical guidelines, improve diagnostic precision, and support effective, evidence-based care that enhances patient outcomes.

Methods: Readability of the content was compared and assessed using the Flesch-Kincaid Reading Ease and Grade Level metrics via an online calculator, evaluating parameters such as word and sentence counts, average sentence length, and proportion of difficult words. Data were compiled in Excel (Microsoft Corp., Redmond, WA, USA), and statistical tools were used for analysis, with significance set at a p-value < 0.05.

Results: In this limited-scope study, ChatGPT (OpenAI, San Francisco, CA, USA)-generated content on pneumonia was found to be significantly shorter and denser than UpToDate, with more complex vocabulary, though both sources showed comparable readability scores across standard metrics.

Conclusion: This suggests ChatGPT may offer quicker, more accessible summaries, while UpToDate provides more balanced, clinically grounded content, highlighting the potential of a combined approach for effective medical education. The clinical accuracy of the AI-generated content was not reviewed by human experts, thus underlining the need for broader studies across diverse clinical topics with multiple reviewers.

## Introduction

Pneumonia, an infection of the lower respiratory tract, causes alveolar accumulation of mucus and pus and commonly presents with cough, fever, and tachypnea [1]. Globally, pneumonia continues to impose a heavy toll on public health [2]. Timely diagnosis and management of pneumonia is very important, and therefore, medical education on the diagnosis, treatment guidelines, and potential complications of this condition is critical. UpToDate is an evidence-based clinical decision support tool authored by over 7,100 physicians. This expert network continuously updates medical recommendations across specialties to assist clinicians with point-of-care decisions [3]. Artificial intelligence utilizes computational systems to replicate cognitive functions typically associated with human thought. The integration of AI within educational settings has expanded steadily since 2018. AI has great potential to advance medical training, improve diagnosis, and impact the future of healthcare workers. However, AI faces several challenges. A major concern with AI systems is the inconsistency in the accuracy and reliability of their outputs, which can at times be incorrect and complicate healthcare decision-making. A well-publicized case is IBM Watson for cancer treatment, which recently faced criticism for allegedly making “unsafe and incorrect” recommendations regarding cancer therapies [4]. Additional concerns include biases against specific groups, biased objectives, and the inability of AI systems to address the bio-psychosocial complexity of patients effectively [4]. Developed by OpenAI, ChatGPT (OpenAI, San Francisco, CA, USA) is a large language model built upon the generative pre-trained transformer (GPT) architecture. It was trained on extensive datasets of internet text [5]. The objective of this study was to compare the readability and linguistic characteristics of educational content on pneumonia generated by ChatGPT with those from UpToDate.

## Materials and methods

Study design and setting

This study was designed as a cross-sectional comparative analysis conducted over one week, from 27th May to 2nd June 2025. The objective was to compare the readability and linguistic characteristics of educational content generated by ChatGPT on pneumonia with reference material from UpToDate, a widely used evidence-based clinical decision support tool. Because the work relied solely on secondary data in the form of textual outputs, without the involvement of human participants or interventions, approval from an Institutional Ethics Committee was not required.

Selection of content

The study examined educational material intended for medical professionals. For the AI component, content was generated using ChatGPT version 4o, accessed between 1st and 2nd June 2025. Six topics were carefully formulated to cover a spectrum of pneumonia-related clinical scenarios: 1) treatment of community-acquired pneumonia in adults in the outpatient setting; 2) treatment of community-acquired pneumonia in adults who require hospitalization; 3) treatment of hospital-acquired and ventilator-associated pneumonia in adults; 4) community-acquired pneumonia in children (outpatient treatment); 5) pneumonia in children (inpatient treatment); and 6) treatment and prognosis of nonspecific interstitial pneumonia. The responses to these six topics were analyzed. For comparison, reference content was retrieved from UpToDate on 27th May 2025. To ensure uniformity, only the main narrative text was included, while tables, figures, references, and hyperlinks were excluded.

Variables and outcomes

The primary outcome of interest was the readability of the content. Readability was assessed using several validated indices, including the Flesch reading ease (FRE) score, the Flesch-Kincaid grade level (FKGL), and the simple measure of gobbledygook (SMOG) index, established readability formulas that are in the public domain and widely used in academic and governmental research and are not subject to copyright protection and do not require permission for use or publication [6]. Additional variables included word count, sentence count, average words per sentence, and both the absolute number and the percentage of difficult words, as defined by the readability calculator algorithm [6]. Together, these measures allowed for the evaluation of both the structural and linguistic complexity of the texts.

Data sources and measurement

Text generated by ChatGPT was copied into Microsoft Word (Microsoft Corp., Redmond, WA, USA) documents, while the UpToDate text was reformatted similarly for consistency. All readability scores were calculated using a standardized online Flesch-Kincaid calculator to ensure uniformity. Raw values, including word and sentence counts, were entered into Microsoft Excel (Microsoft Corp.) spreadsheets. To reduce error, content extraction and readability calculations were verified independently by two authors.

Bias control

To minimize bias, identical clinical topics were selected for both sources. Prompts for ChatGPT were standardized to request educational guides specifically tailored for medical professionals. By excluding non-textual material from UpToDate, the study ensured that comparisons were made solely on narrative text. Independent verification by two reviewers further reduced the potential for measurement error.

Study size and rationale

A total of six distinct pneumonia-related topics were included in the analysis. These topics were chosen to represent a range of clinical settings spanning adult and pediatric populations, outpatient and inpatient care, and hospital-acquired conditions. The study size was considered appropriate for exploratory analysis, although the limited scope of one disease area was acknowledged as a restriction on broader generalizability.

Statistical analysis

All statistical analyses were conducted using SPSS Statistics version 25 (IBM Corp., Armonk, NY, USA) and R software version 4.3.2 (R Foundation for Statistical Computing, Vienna, AUT). Continuous variables were summarized as medians with interquartile ranges. Comparisons between ChatGPT and UpToDate outputs were performed using the Mann-Whitney U test. A two-tailed p-value of less than 0.05 was considered statistically significant. Findings are presented in both tabular and graphical formats to facilitate interpretation and to highlight differences in readability metrics between the two sources.

## Results

ChatGPT and UpToDate were used to generate educational content for medical professionals on pneumonia. The responses were evaluated based on readability parameters, including word count, sentence count, word/sentence ratio, FRE, FKGL, SMOG index, difficult word count, and difficult word percentage. The aim was to assess whether AI-generated material matched the readability and complexity of content from a trusted clinical reference. A comparison of readability metrics between ChatGPT and UpToDate is presented in Table 1.

**Table 1:** Characteristics of responses generated by UpToDate and ChatGPT The analysis was performed using SPSS Statistics version 25 and R version 4.3.2. Mann-Whitney’s U Test was used to compare the distribution of responses generated by UpToDate and ChatGPT. Based on the p-values obtained in Table 1, there is a statistically significant difference between the median word count, sentence count, difficult word count, and difficult word percentage generated by the two AI tools. FRE: Flesch reading ease; FKGL: Flesch-Kincaid grade level; SMOG: Simple measure of gobbledygook; ^+^Mann-Whitney’s U test; A p-value <0.05 is considered statistically significant.

Parameters	Median (IQR)		U statistic	p-value
	UpToDate	ChatGPT		
Word count	3134.0 (1013.2 – 5718.8)	218.0 (185.7 – 430.5)	0	0.004*
Sentence count	175.0 (51.5 – 325.0)	27.5 (10.5 – 38.5)	2	0.010*
Word/sentence count	18.2 (16.4 – 20.4)	11.2 (7.7 – 9.4)	6	0.055
FRE	14.1 (10.8 – 18.8)	17.3 (9.4 – 20.9)	15	0.631
FKGL	15.8 (15.2 – 16.6)	13.8 (12.7 – 16.4)	6	0.054
SMOG index	13.4 (12.3 – 13.8)	10.8 (9.9 – 12.9)	6	0.055
Difficult word count	952.0 (293.2 – 1605.2)	81.0 (62.2 – 148.5)	2	0.010*
Difficult word percentage	28.6 (27.0 – 29.9)	34.0 (29.4 – 37.6)	5	0.037*

**Figure 1:**
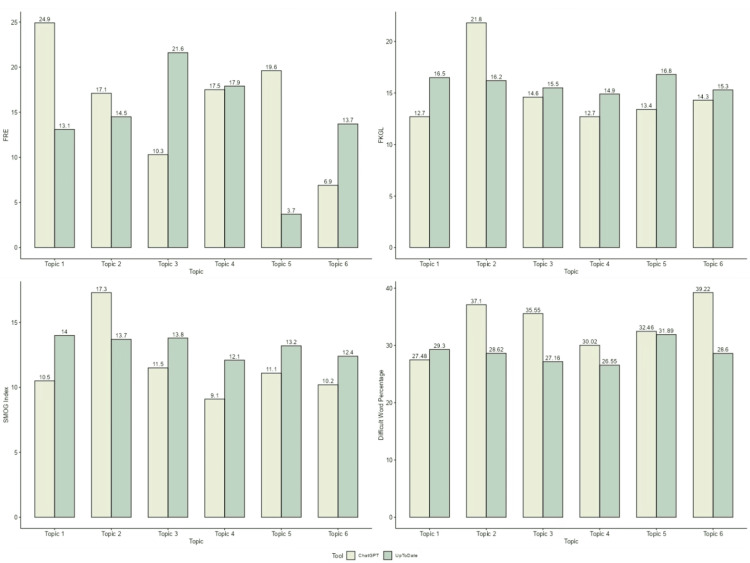
Graphical representation of comparison between FRE, FKGL, SMOG index and percentage of difficult words for the patient education guide generated by UpToDate and ChatGPT FRE: Flesch reading ease; FKGL: Flesch-Kincaid grade level; SMOG: Simple measure of gobbledygook Topic 1: Treatment of community-acquired pneumonia in adults in the outpatient setting; Topic 2: Treatment of community-acquired pneumonia in adults who require hospitalization; Topic 3: Treatment of hospital-acquired and ventilator-associated pneumonia in adults; Topic 4:  Community-acquired pneumonia in children (outpatient treatment); Topic 5: Pneumonia in children (inpatient treatment); Topic 6: Treatment and prognosis of nonspecific interstitial pneumonia

There was a statistically significant difference in word count (p = 0.004), sentence count (p = 0.010), and percentage of difficult words (p = 0.037), with ChatGPT responses being significantly shorter and denser, yet containing a higher proportion of complex vocabulary. No statistically significant differences were found in average words per sentence (p = 0.055), FRE (p = 0.631), FKGL (p = 0.149), or SMOG index (p = 0.078), suggesting comparable readability levels in those metrics. These findings imply that while ChatGPT content may be quicker to read, the higher vocabulary complexity could be challenging for healthcare workers with varying levels of training, especially in lower-resource settings. Based on the p-values (Table 1), there is a statistically significant difference between the median word count, sentence count, difficult word count, and difficult word percentage generated by the AI tools. ChatGPT produced shorter and more condensed sentences in comparison to UpToDate, as seen by the comparison of word and sentence counts. ChatGPT information showed a higher difficulty percentage but a lower number of difficult words and a higher FRE score in comparison to UpToDate. Figure 1 provides a visual comparison of the readability metrics FRE, FKGL, SMOG index, and difficult word percentage for content generated by ChatGPT and UpToDate across six pneumonia topics.

The FRE scores were consistently higher for ChatGPT across most topics, suggesting easier readability. The largest difference was observed in topic five (ChatGPT: 19.6 vs. UpToDate: 3.7). However, ChatGPT content also showed a consistently higher difficult word percentage (e.g., topic two: ChatGPT 37.1% vs. UpToDate 28.62%), indicating the use of more complex terminology despite a simpler sentence structure. For healthcare workers, this contrast means that ChatGPT can be a useful tool for generating quick, accessible content ideal for handouts, on-the-spot education, or rapid review, while UpToDate offers in-depth, clinically validated resources better suited for comprehensive learning and clinical reference. The FKGL and SMOG index values showed minor variation between the two sources. In most topics, ChatGPT had slightly lower grade levels. A combined approach could leverage the speed and simplicity of ChatGPT with the depth and accuracy of UpToDate to enhance educational efficiency without compromising quality.

## Discussion

This cross-sectional study compared educational content generated by ChatGPT with UpToDate for medical professionals on the treatment of community-acquired pneumonia. Artificial intelligence tools, such as ChatGPT, can potentially aid learning by providing concise medical information to students quickly and efficiently and may even include interactive simulations [7]. ChatGPT increases student involvement and improves students' ability to learn independently, allowing students to depend less on textbooks [8]. Artificial intelligence tools empower every early-career professional, trainee, student, and clinician to undertake more research, allowing better learning and deeper understanding of health systems [9].

Readability scores such as the FRE and SMOG Index are used to evaluate how easy it is to understand medical text. The study by Zhou et al. shows that ChatGPT and DeepSeekAI (DeepSeek, Hangzhou, CHN) produce information with higher readability scores. They also state that enhanced readability can improve patient engagement and contribute to better surgical outcomes [10]. In this study, ChatGPT had higher FRE scores and lower difficult word counts than UpToDate, suggesting it may be easier to read, even for a clinical audience, particularly under time constraints.

In a study done by Erden et al., it was found that ChatGPT information was easier to obtain for topics like osteoporosis, but the quality and readability of the information did not meet healthcare standards [11]. In another study done by Carlson et al., it was concluded that five AI chatbots used to describe urology-specific medical information on average had FRE scores less than 50 and were greater than a 10th-grade level [12]. Similar patterns were seen in structure, readability, and language complexity over multiple studies. Other studies also noted moderate readability and variation in text clarity. Common trends suggest AI tools perform consistently across different topics. This reinforces the potential of AI in supporting medical education.

The results of Temel et al.'s study showed heightened complexity in ChatGPT-generated texts on spinal cord injury, which surpassed optimal health communication readability [13]. In another study, ChatGPT demonstrated its capability to address radiotherapy concepts. However, it presented readability challenges for the general population [14]. Another study by Behars et al. shows that AI chatbots can answer medical questions on topics such as cardiac catheterization. The responses using four chatbots in the study had a mean reading grade level of 11th to 13th grade, depending on the tool used, which exceeds the sixth-grade level for patient education materials [15]. Some studies found AI content overly simplified or lacking clinical depth. Differences may be due to the AI version, type of prompt, or topic chosen. Variability in evaluation methods or the reviewer's perspective could also play a role. This issue emphasizes that there must be standardized assessments of AI-generated content.

Limitations

This study has several limitations. First, it assessed only one AI tool (ChatGPT-4o) and a single disease area (pneumonia). The findings may therefore not be generalizable to other AI models or clinical topics. Second, the evaluation was restricted to readability and linguistic characteristics using standardized indices (FRE, FKGL, SMOG index, difficult word count/percentage), which, although widely applied, are originally designed for general audiences and may not fully capture the nuances of medical professional texts. Third, the study did not evaluate the clinical accuracy, reliability, or comprehensiveness of the AI-generated content, which are critical for determining its suitability in medical education and practice. Fourth, reproducibility is limited since AI outputs can vary with version updates, prompt phrasing, or server conditions. The prompts used in this study are featured in Appendix A to improve transparency; however, future replication may yield different results. Finally, the relatively small sample size (six topics) and focus on narrative text alone further constrain the generalizability of these findings.

## Conclusions

This cross-sectional study compared the readability and linguistic complexity of educational content on pneumonia generated by ChatGPT and UpToDate. Statistically significant differences were found in word count, sentence count, and difficult word percentage, with ChatGPT producing shorter, denser text that contained a higher proportion of complex vocabulary. Despite these structural differences, overall readability scores (FRE, FKGL, and SMOG index) were not significantly different between the two sources. These findings suggest that ChatGPT has the potential to provide concise summaries or rapid educational guides, while UpToDate remains a more comprehensive, clinically validated reference. However, because this study did not evaluate clinical accuracy, conclusions must be interpreted strictly within the scope of readability analysis. Broader studies, incorporating multiple AI tools, diverse clinical topics, and formal expert review for accuracy, are necessary to determine the true educational and clinical value of AI-generated medical content.

## Appendices

Appendix A

The prompts provided to ChatGPT (model 4o) in this study are as follows: “Generate an educational guide for medical professionals on the outpatient treatment of community-acquired pneumonia in adults”; “generate an educational guide for medical professionals on the inpatient treatment of community-acquired pneumonia in adults who require hospitalization”; "generate an educational guide for medical professionals on the treatment of hospital-acquired pneumonia and ventilator-associated pneumonia in adults"; "generate an educational guide for medical professionals on the outpatient management of community-acquired pneumonia in children"; "generate an educational guide for medical professionals on the inpatient management of community-acquired pneumonia in children"; and "generate an educational guide for medical professionals on the treatment and prognosis of nonspecific interstitial pneumonia”.
